# Adenovirus‐Specific T Cells in Adults Are Frequent, Cross‐Reactive to Common Childhood Adenovirus Infections and Boosted by Adenovirus‐Vectored Vaccines

**DOI:** 10.1002/jmv.70222

**Published:** 2025-02-08

**Authors:** Rookmini Mukhopadhyay, Arnold W. Lambisia, Jennifer P. Hoang, Benjamin J. Ravenhill, Charles N. Agoti, Benjamin A. Krishna, Charlotte J. Houldcroft

**Affiliations:** ^1^ Department of Genetics University of Cambridge Cambridge UK; ^2^ Kenya Medical Research Institute‐Wellcome Trust Research Programme Kilifi Kenya; ^3^ Cambridge Institute for Medical Research, School of Clinical Medicine University of Cambridge Cambridge UK; ^4^ Department of Medicine University of Cambridge Cambridge UK

**Keywords:** cellular immunity, DNA virus, vaccination, viral vectors

## Abstract

Human adenoviruses (HAdVs) cause diverse disease presentations as pathogens and are also used as viral vectors for vaccines and gene therapy products. Pre‐existing adaptive immune responses to HAdV are known to influence symptom severity, viral clearance and the success of viral vectored products. Of note, approximately 50% of the UK's adult population has received at least one dose of a chimpanzee adenovirus vectored SARS‐CoV‐2 vaccine (ChAdOx1) since January 2021. We used FluoroSpot analysis to quantify the interferon‐gamma (IFNγ) and interleukin‐2 (IL2) responses of healthy blood donors to HAdV species A, B, C, D and F and chimpanzee adenovirus Y25, related to HAdV species E. We find that cellular immune responses to multiple species of human adenovirus are ubiquitous among healthy adult blood donors and that stimulating PBMC with whole hexon peptide libraries induces a significantly greater IFNγ and IL2 response than using selected peptide pools alone. We then compared the cellular immune responses of ChAdOx1 recipients and control donors using PBMC collected in 2021 and found that homotypic and heterotypic IFNγ responses were significantly boosted in ChAdOx1 recipients but not controls. Finally, we show that in PBMC derived from blood donors, IFNγ responses are made to both conserved and variable regions of the hexon protein. Future vaccination campaigns using adenoviral vectored vaccines will need to account for the pre‐existing exposure of recipients to both circulating HAdVs and vaccines such as ChAdOx1, which convey polyfunctional antiviral T cell responses to even low seroprevalence HAdV types.

**Abbreviations**
ChAd: chimpanzee adenovirus
ELISA: enzyme‐linked immunosorbent assay
FluoroSpot: fluorescence‐linked immunosorbent spot
HAdV: human adenovirus
HRA: Health Research Authority
IFNγ: interferon gamma
IL2: interleukin 2
nAbs: neutralising antibodies
NHSBT: National Health Service Blood & Transplant
PBMC: peripheral blood mononuclear cells
SARS‐CoV‐2: severe acute respiratory syndrome coronavirus 2
SFU: spot forming units

## 1 Introduction

Adenoviruses (AdVs) are non‐enveloped, double‐stranded DNA viruses with icosahedral capsids comprised of three proteins: hexon, penton and fibre [1]. More than 100 human serologically or genotypically distinct types of adenoviruses (HAdVs) have been identified to date, and classified into seven species (A to G). The majority of primary HAdV infections occur during the first 5 years of life and cause symptoms ranging from upper and lower respiratory tract infections and keratoconjunctivitis, to gastro‐intestinal disease and fulminant infection [2]. Currently, there is no approved treatment for adenovirus infection, and no vaccine available for civilian use [3, 4]. Different HAdV species are associated with different kinds of disease, and genetic recombination in the capsid genes of different AdVs can lead to altered tissue tropism and symptom profiles [5].

There has been significant interest in the immune response against HAdVs, largely driven by the challenge of pre‐existing HAdV immunity against HAdV‐derived vectors, which suppresses the efficacy of immunisation [6]. This is likely due to a biasing of immune responses to memory responses against the AdV backbone rather than de novo responses to the vaccine antigen. Previous studies have primarily focused on HAdV‐C5. Passive neutralising antibody (nAb) transfer experiments in naïve mice dampened immune stimulation by HAdV‐C5 vectors but to a lesser extent than the dampening observed in pre‐immune mice. Therefore nAbs alone cannot account for all pre‐existing immunity, implicating AdV‐specific T cells in the AdV‐induced immune response [7]. Passive transfer of CD8^+^ T cells into naïve mice significantly decreased the immune response induced by HAdV‐C5 vectors, highlighting their role in immune dampening [7]. The role of T cells in adenoviral clearance is illustrated by the success of adoptive T‐cell therapy, where HAdV‐infected patients are treated by transfusion of HAdV‐specific T cells, with an overall 75% response rate [8]. These data reinforce the importance of T cells in resolving HAdV infection in healthy and immunocompromised individuals and their potential role in dampening the immunisation efficacy of HAdV‐derived vectors due to cross‐reactivity.

**Figure 1:**
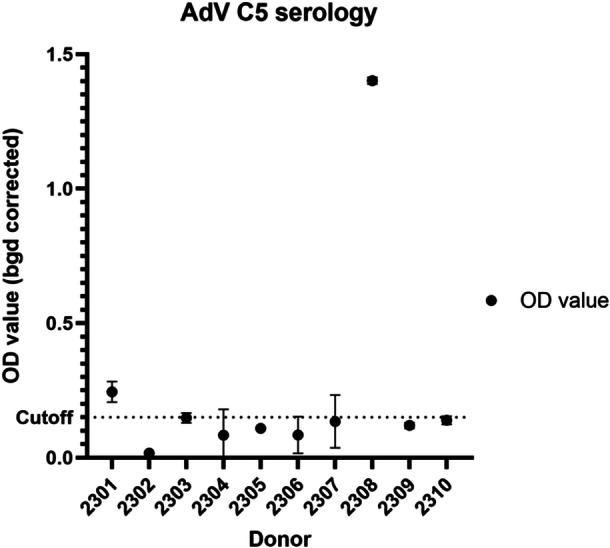
Plot showing serum antibody responses to adenovirus C5 in healthy blood donors. Optical density measurements were made using an ELISA specific for HAdV C5 and were background‐corrected.

Previous studies have suggested that HAdV‐specific T cells are cross‐reactive, capable of recognising a broad range of HAdVs. This is exemplified by the ability of HAdV‐specific T cells to recognise chimpanzee‐derived AdVs [9]. Cross‐reactivity arises from the ability of T cells to recognise conserved peptide regions, usually in the hexon [10], or in nonstructural proteins such as the DNA polymerase [11] and Early Region 1A (E1A) [12]. MHC‐II‐restricted CD4^+^ epitopes have been identified throughout the hexon, but predominantly in the conserved regions [9, 10, 13], while MHC‐I‐restricted CD8^+^ T cell epitopes have also been identified in the hexon, penton and fibre [10, 14, 15] (Supporting Information S1: Figure 1) T cell responses to conserved regions are presumably recalled repeatedly upon infection with different HAdVs, whereas responses to variable regions are less frequently stimulated [9]. By contrast, nAbs show limited cross‐neutralisation of different HAdV subtypes, which is a consequence of nAbs targeting peptide regions with high heterogeneity, such as the hexon hypervariable regions (HVRs) [16, 17, 18].

In addition to the cellular immune landscape of natural HAdV infection, approximately 50% of the UK's adult population have received ChAdOx1, the Oxford‐Astra Zeneca SARS‐CoV‐2 vaccine [19]. This is a viral‐vectored vaccine which is based on chimpanzee adenovirus Y25 (GenBank accession: JN254802) and contains a deletion of Y25 genes E4, ORFs 4, 6, 7 and the 34 K CDS region and insertion of the equivalent portion of the human adenovirus C5 genome [20, 21].

We therefore set out to investigate the interferon‐gamma (IFNγ) and interleukin 2 (IL2) T cell response of healthy blood donors to diverse human AdVs from multiple species; to establish whether the T cell response to a non‐species C human adenovirus was confined to the conserved regions of the hexon protein; and to quantify what effect the use of ChAdOx1 has had on the landscape of anti‐adenovirus T cell immunity in UK donors.

## 2 Methods

### 2.1 Donors

#### 2.1.1 Healthy Blood Donors

Samples from 10 anonymised healthy blood donors were collected from NHS Blood and Transplant (NHSBT), Cambridge Donor Centre. Ethical permission for ‘Understanding humoral and cellular immune responses to DNA viruses in healthy blood donors’ was granted by the HRA and Health and Care Research Wales (HCRW) (REC reference [22]/WA/0162). Platelet donors from whom leucocyte cones were derived are aged between 17 and 70; specific age and sex data for individual donors were not available.

Thirty‐two donors of known SARS‐CoV‐2 vaccine status were recruited in 2021, all patients gave informed written consent in accordance with the Declaration of Helsinki. Ethical permission for the ARIA (Antiviral Responses in Ageing, CBR53) study was granted by the Cambridge Human Biology Research Ethics Committee (HBREC.2014.07). Donors were grouped as recipients of one or more doses of ChAdOx1 vaccine (ChAdOx1 recipients), *n* = 17; or one or more doses of mRNA vaccine or no vaccine at the point of blood donation (controls), *n* = 15. Donors are aged between 18 and 65. Participants were excluded from the study if they received oral or intravenous immunomodulatory drugs (including steroids or immunosuppressants) within the past 3 months, received injected anti‐TNF treatments for rheumatoid arthritis, or received current or recent (past 24 months) cancer chemotherapy.

### 2.2 Serology

In blood donors, IgG responses to HAdV‐C5 were measured by ELISA using a Human Adenovirus IgG (ADV‐IgG) ELISA Kit [AE24150HU] (Abebio, Wuhan, China), following the manufacturer's recommended protocol. For samples 2301‐2304, haemolysate was used to counter erythrocyte contamination due to NHSBT cone storage duration of longer than 12 h before serum sampling; for samples 2305–2310, serum was used.

### 2.3 Peptide Stimulants

#### 2.3.1 Adenovirus ORF and Other Peptide Mixes

Six commercially available, and two custom, peptide pools were selected to represent the diversity of human adenovirus species (Table 1). Commercial peptide pools from Miltenyi and JPT (Table 1) were diluted to a concentration of 5 μg/mL/peptide. A custom library of consecutive 15‐mer peptides overlapping by 5 amino acids was synthesised by GenScript (Oxford, UK) using the HAdV‐A12 hexon sequence (GenBank accession: NP_040924.1). Individual lyophilised peptides from each custom ORF library were reconstituted in 20% DMSO‐80% RPMI‐1640 (Sigma) at 10 mg/mL master stock. Individual peptides were then diluted in RPMI‐1640 to give a 1 mg/mL (2% DMSO) working stock. Peptide pools were used as either entire ORF mixes at a concentration of 5 μg/mL/peptide (final working concentration shown in Table 1) or formed into pools of 40–60 peptide pool of conserved and variable epitopes (see below), at a concentration of 20 μg/mL/peptide.

**Table 1:** Proteins and peptide pools used for T cell stimulation.

Name	Supplier	Adenovirus species and type	Final concentration
Custom HAdV A12 hexon pool	GenScript	A: 12	2 μg/ml/peptide
HAdV‐A conserved and variable pools	GenScript	A: 12	20 μg/mL/peptide
PepMix HAdV‐3 hexon (PM‐HAdV3)	JPT	B: 3	2 μg/mL/peptide
PepTivator AdV Select (130‐124‐394)^a^	Miltenyi	C: 2 and 5	2 μg/mL/peptide
PepTivator AdV5 Hexon (130‐093‐495)	Miltenyi	C: 5	2 μg/mL/peptide
PepTivator AdV5 Penton (130‐096‐777)	Miltenyi	C: 5	2 μg/mL/peptide
PepMix Human Adenovirus 26 Hexon (PM‐HADV26‐L3‐1)	JPT	D: 26	2 μg/mL/peptide
PepMix Human Adenovirus 26 Penton (PM‐HADV26‐L2‐1)	JPT	D: 26	2 μg/mL/peptide
Custom HAdV F41 hexon pool	GenScript	F: 41	2 μg/mL/peptide
PepMix Chimpanzee Adenovirus Y25 Hexon (PM‐CHADVY25‐L3‐1)	JPT	ChAd: Y25	2 μg/mL/peptide
PepMix Chimpanzee Adenovirus Y25 Penton (PM‐CHADVY25‐L2‐1)	JPT	ChAd: Y25	2 μg/mL/peptide

a AdV Select is a pool of 50 MHC class I and class II restricted oligopeptides derived from human adenovirus species C genotypes 2 and 5.

#### 2.3.2 Defining Conserved Versus Variable Regions

Peptides were defined as conserved if there were eight consecutive amino acids out of 15 identical between the C5 and A12 hexon amino acid reference sequences (AP_000211.1 and NP_040924.1 respectively). The epitopes constituting the A12 conserved 1, conserved 2 and variable pools are listed in Supporting Information S1: Table 1.

PBMC and serum isolation, and detection of cytokine production in PBMC by FluoroSpot were performed as previously described [22] and detailed in Supplementary Methods. Multiple sequence alignment and amino acid distance calculations are described in Supplementary Methods.

### 2.4 Statistical Analyses

Data were tested for normality using Shapiro–Wilk tests and parametric or non‐parametric statistical tests chosen accordingly. Spearman's R was calculated for the averaged background corrected SFU per 10^6^ PBMC for each pair of hexon proteins. SFU which were 0 after background correction are represented as 0.1 on log scale graphs. Sample size power calculations were performed as described in [23] and detailed in Supplementary Methods. Unless otherwise stated, statistical analysis and graphing were performed in GraphPad Prism v10.3. A Mantel test of the relationship between hexon pairwise amino acid distances and IFNγ response correlations was performed in Matlab 2021b, using the Fathom toolbox [24], set at 9999 permutations.

## 3 Results

### 3.1 Serological Responses to Adenovirus C5 Are Relatively Uncommon

Serum antibody responses to adenovirus are often used as a proxy for previous infection history [25]. We therefore measured anti‐adenovirus C5‐IgG levels by ELISA in each of our healthy donors (Figure 1). 2/10 donors had a positive IgG response to adenovirus C5, and 4/10 had equivocal responses (confidence intervals overlapping the cutoff). The remaining four donors were seronegative. This is broadly in line with binding antibody data collected in Germany [26, 27], where binding antibody levels were higher for genotype C1 than C5, and for previous serology surveys conducted in Europe [25]. This may also mirror the significant post‐COVID reduction in binding antibody levels seen in German healthy serum donors between 2019 and 2021 [27], but the numbers presented here are small. No comparable data is available for England.

### 3.2 Interferon Gamma‐Producing Responses to Adenoviruses Are Pervasive and IL2 Responses Are Seen in the Majority of Healthy Donors

**Figure 2:**
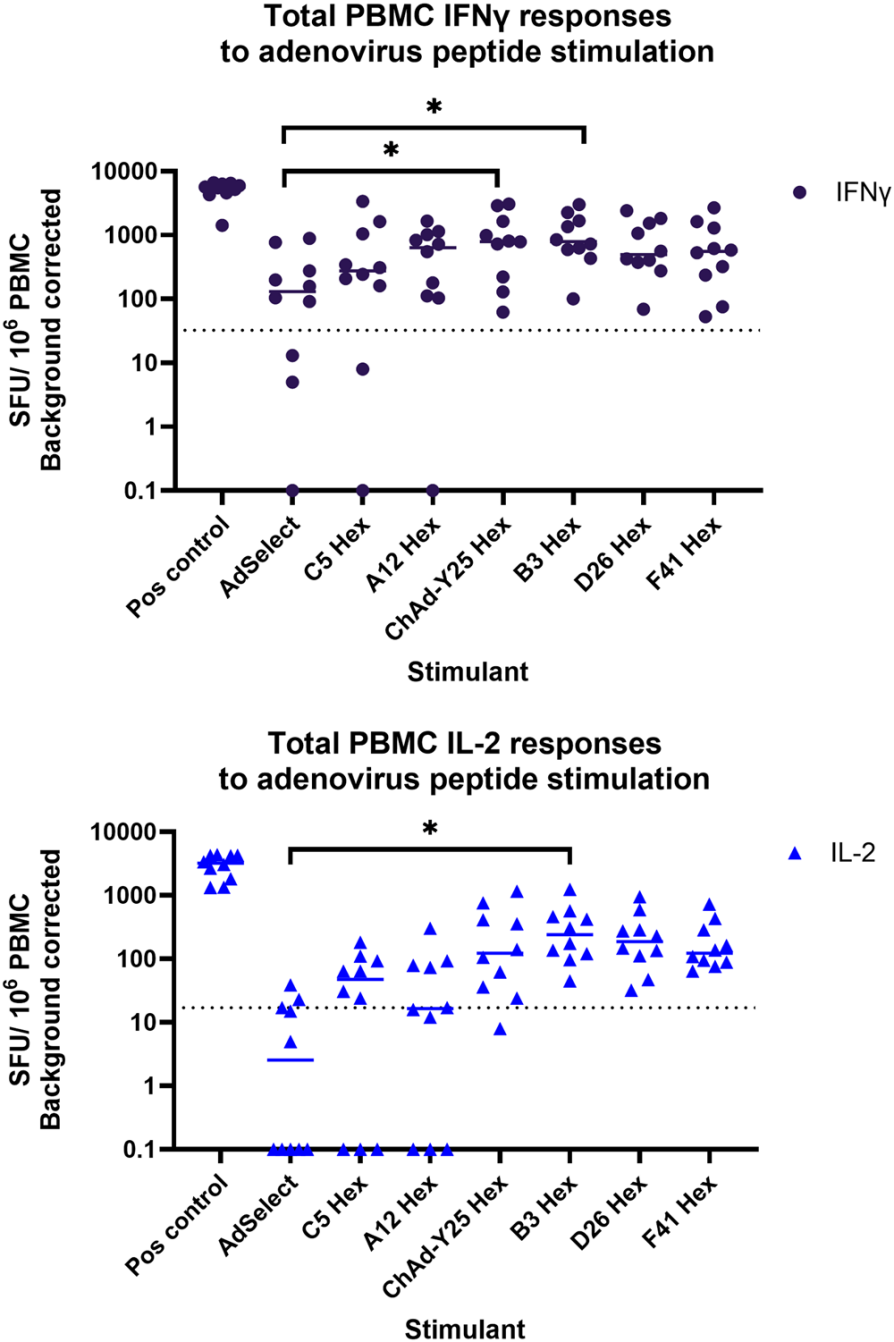
Analysis of AdV specific IFNγ and IL2 FluoroSpot responses to AdV peptide pools (A) IFNγ FluoroSpot responses to AdV peptide pools covering: an experimentally‐validated set of 50 AdV C2 and 5 peptides (AdSelect), peptide pools covering the entire hexons of human adenoviruses A12, B3, C5, D26, F41 and the hexon of chimpanzee adenovirus Y25 (vector backbone of SARS‐CoV‐2 vaccine ChAdOx1), as well as a polyclonal anti‐CD3/CD28 antibody T cell stimulation as a positive control of PBMC from healthy blood donors, calculated as spot‐forming units (SFU) per 10^6^ PBMC (background corrected). (B) IL2 FluoroSpot responses to AdV peptide pools covering: an experimentally‐validated set of 50 AdV C2 and 5 peptides (AdSelect), pools covering the entire hexons of human adenoviruses A12, B3, C5, D26, F41, the hexon of chimpanzee adenovirus Y25 (vector backbone of SARS‐CoV‐2 vaccine ChAdOx1), as well as a polyclonal anti‐CD3/CD28 antibody T cell stimulation as a positive control of PBMC from healthy blood donors, calculated as spot‐forming units (SFU) per 10^6^ PBMC (background corrected). Significance determined by two‐way ANOVA, corrected for multiple testing. Key: *p < 0.05. The dotted lines indicate the boundary between a positive and a negative response.

**Figure 3:**
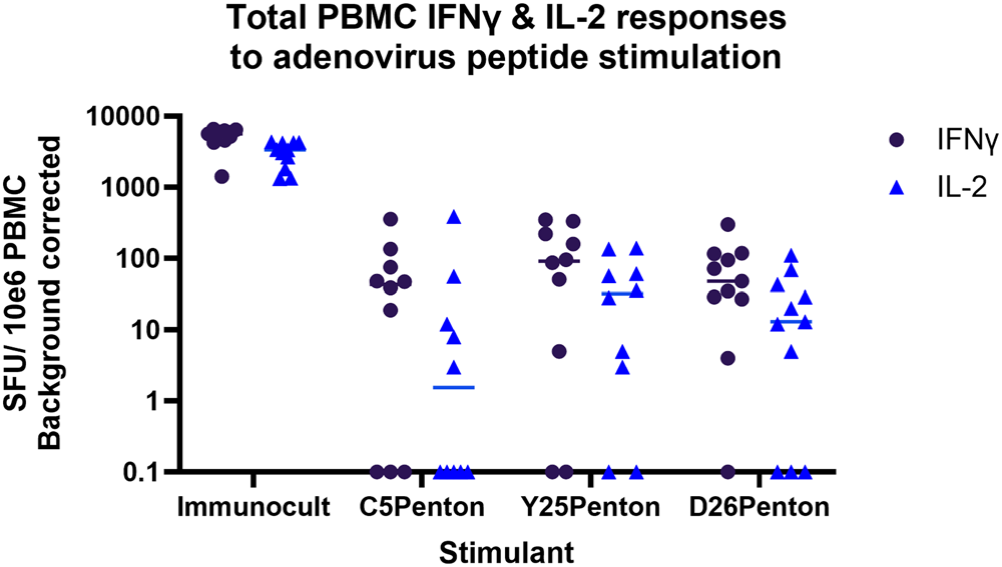
Analysis of AdV specific IFNγ and IL2 FluoroSpot responses to AdV peptide pools (A) IFNγ FluoroSpot responses to AdV peptide pools covering: the pentons of human adenoviruses C5 and D26, and the penton of chimpanzee adenovirus Y25 (vector backbone ChAdOx1) as well as a polyclonal anti‐CD3/CD28 antibody T cell stimulation as a positive control of PBMC from healthy blood donors, calculated as spot‐forming units (SFU) per 10^6^ PBMC (background corrected). (B) IL2 FluoroSpot responses to AdV peptide pools covering: the pentons of human adenoviruses C5 and D26, and the penton of chimpanzee adenovirus Y25 (vector backbone ChAdOx1) as well as a polyclonal anti‐CD3/CD28 antibody T cell stimulation as a positive control of PBMC from healthy blood donors, calculated as spot‐forming units (SFU) per 10^6^ PBMC (background corrected).

IFNγ responses, measured by FluoroSpot or similar technologies, are an in vitro proxy for in vivo antiviral T cell recognition and response to viral peptides. T cell responses are an alternative tool for identifying previous viral pathogen exposure in seronegative individuals [23]. Cutoffs for positivity were determined based on analysis of responses to the Y25 hexon peptide pool in adenovirus‐vectored vaccine recipients and controls (Supporting Information S1: Figure 2, supplementary material). Using peptides derived from prevalent adenovirus species C genotypes, we found that 7/10 donors made a positive IFNγ response to the AdV Select (C2 and C5) pool, 8/10 to the C5 hexon peptide pool and 9/10 to the A12 hexon peptide pool (Figure 2). The AdV Select pool is a set of peptides spanning epitopes experimentally identified from genotypes 2 and 5, while the C5 hexon pool tiles the whole of the immunodominant hexon protein. All donors made a positive IFNγ response to the hexons of HAdVs B3, D26 and F41, demonstrating clear exposure to a range of HAdV species even in the absence of detectable antibody serology. There was a statistically significant correlation in within‐donor mean IL2 and IFNγ responses for all peptide pools, with the exception of the AdV select commercial peptide pool (Supporting Information S1: Figure 3).

**Figure 4:**
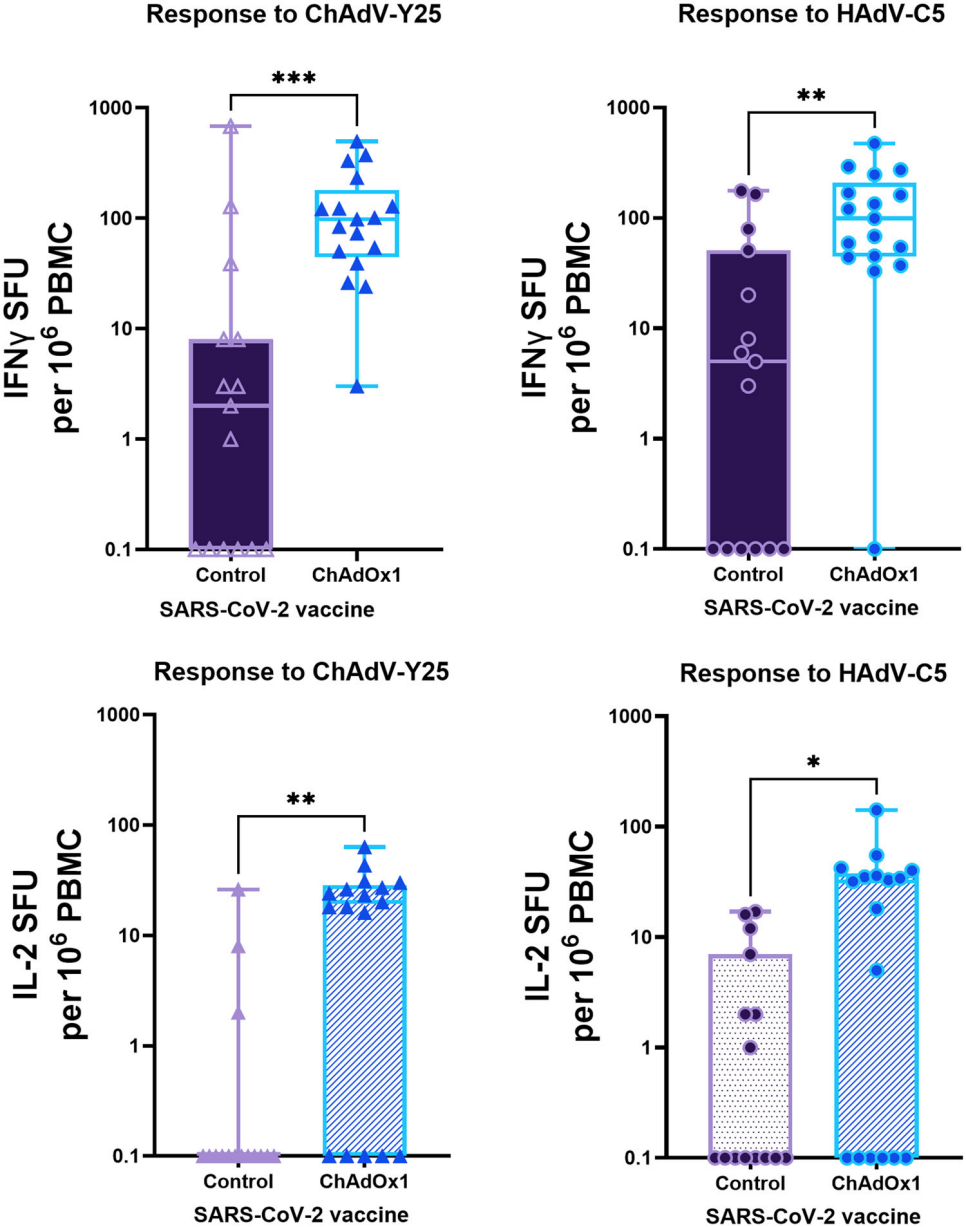
Frequency of IFNγ (A) and IL2 (B) responses to adenovirus peptide pools covering the hexon proteins of Y25 and C5 in ChAdOx1 vaccine recipients and controls (mRNA vaccine or no vaccine), expressed as spot‐forming units per 10^6^ PBMC (background corrected). IFNγ responses are shown as filled bars. IL‐2 responses are shown as bars with horizontal stripes. Mean individual responses to ChAdV‐Y25 are shown as triangles. Mean individual responses to HAdV‐C5 are shown as circles. Control recipients are shown in purple, ChAdOx1 recipients in blue. *P* values were calculated with a Mann–Whitney U test.

IL2 responses are a proxy for CD4^+^ T cell recognition of viral peptides [28]. Similar to IFNγ, 7/10 donors had a positive IL2 response to the C5 hexon pool. However, fewer donors made a positive response IL2 (3/10 donors) to the AdV Select pool, compared to IFNγ (7/10). The estimated input CD3^+^ CD4^+^ cell number did not significantly correlate with IL2 SFU responses to the C5 hexon, Y25 hexon or AdV Select pools (Supporting Information S1: Figure 4).

### 3.3 Interferon Gamma and interleukin‐2 Responses to Adenovirus Penton Peptide Pools Are Less Common in Healthy Blood Donors Than Hexon Responses

The adenovirus penton protein is known to be a target of human T cell responses, though the relative contribution of the penton and hexon to the T cell response to adenovirus infection is currently an area of active research [29]. We therefore quantified the IFNγ and IL2 responses to two commercially available human adenovirus penton peptide pools: HAdVs‐C5 and D26. We used positivity cutoff values derived from hexon responses as described above, as clinically derived penton‐specific cutoffs are not currently available.

We found that positive IFNγ responses to the C5 penton were less common than C5 hexon responses, with 6/10 donors responding to the C5 penton peptide pool (Figure 3) compared to 8/10 responding to the hexon pool (Figure 2). The same was also true of D26 (7/10 responding to the penton, compared to 10/10 responding to the hexon).

Similar trends were seen for IL2 responses, with 2/10 donors responding to penton peptide pools derived from C5, and 5/10 responding to D26 (Figure 3).

### 3.4 IFNγ and IL2 Responses to the Hexon of Chimpanzee Adenovirus Y25 Are Ubiquitous Among Healthy Blood Donors Recruited in 2023

In 2021–2022, an estimated 50% of the UK's adult population received at least one dose of an adenovirus‐vectored SARS‐CoV‐2 vaccine, predominantly ChAdOx1 [19]. The ChAdOx1 vector is based on ChAd‐Y25, retaining the ChAd‐Y25 hexon and penton [20, 21]. ChAd‐Y25 has homology to HAdV species E [21], which is associated with outbreaks of respiratory disease in congregate settings [30].

We found that 10/10 healthy blood donors made a positive IFNγ response to the hexon of ChAd‐Y25 and 9/10 donors made a positive IL2 response (Figure 2). Similar to observations of human adenovirus‐derived penton peptide pools, fewer healthy donors made a positive IFNγ (7/10) or IL2 (6/10) response to the Y25 penton (Figure 3) than the hexon.

### 3.5 Positive IFNγ Responses to Y25 and C5 Hexon Peptide Pools Are More Common in ChAdOx1 Vaccine Recipients Than Controls

Among anonymised apheresis cones from healthy blood donors recruited in 2023, ChAdOx1 vaccination status was not available. We hypothesised that among healthy donors of known ChAdOx1 recipient status, ChAdOx1 recipients would have higher T cell responses to the Y25 vector hexon than recipients who had received an mRNA SARS‐CoV‐2 vaccine or no vaccine instead. We also hypothesised that boosting of C5 hexon T cell responses might occur due to vaccine‐induced activation of cross‐reactive T cells which recognised shared epitopes. We therefore compared PBMC responses to the Y25 and C5 hexons in healthy controls and ChAdOx1 recipients of known vaccine status, vaccinated and recruited in 2021.

Positive IFNγ responses to Y25 and C5 hexon peptide pools, > 32.5 SFU/10^6^ cells, were more common among ChAdOx1 SARS‐CoV‐2 vaccine recipients than controls (mRNA SARS‐CoV‐2 vaccine or no vaccine). 13/17 ChAdOx1 recipients had a positive IFNγ response to the Y25 hexon peptide pool while 2/15 controls had a positive response to Y25. 12/17 ChAdOx1 recipients had a positive IFNγ response and 4/15 controls had a positive IFNγ response to the C5 peptide pool. We found that IFNγ T cell responses were statistically significantly higher in ChAdOx1 recipients for both their responses to the Y25 hexon (mean SFU/10^6^ cells 138.6, SD 139.1 vs mean SFU/10^6^ cells 58.37, SD 176.0; Mann–Whitney U test (two‐tailed) *p* = 0.0003) and the C5 hexon (mean SFU/10^6^ cells 135.7, SD 123.6 vs mean SFU/10^6^ cells 34.17, SD 59.60; Mann–Whitney U test (two tailed) *p* = 0.0015) peptide pools (Figure 4A).

IL2 responses were also more common among ChAdOx1 recipients and controls. 12/17 ChAdOx1 recipients had a positive IL2 response to the Y25 hexon peptide pool, > 17 SFU/10^6^ cells, while 1/15 controls had a positive response. 10/17 ChAdOx1 recipients and 3/15 controls had a positive IL2 response to the C5 hexon peptide pool. The mean SFU was statistically significantly higher for both Y25 (mean SFU/10^6^ cells 19.97, SD 17.16 vs mean SFU/10^6^ cells 2.48, SD 6.82; Mann–Whitney U test (two tailed) *p* = 0.0014) and C5 (mean SFU/10^6^ cells 27.74, SD 34.75, vs mean SFU/10^6^ cells 3.85, SD 6.12; Mann–Whitney U test (two tailed) *p* = 0.023) (Figure 4B).

**Figure 5:**
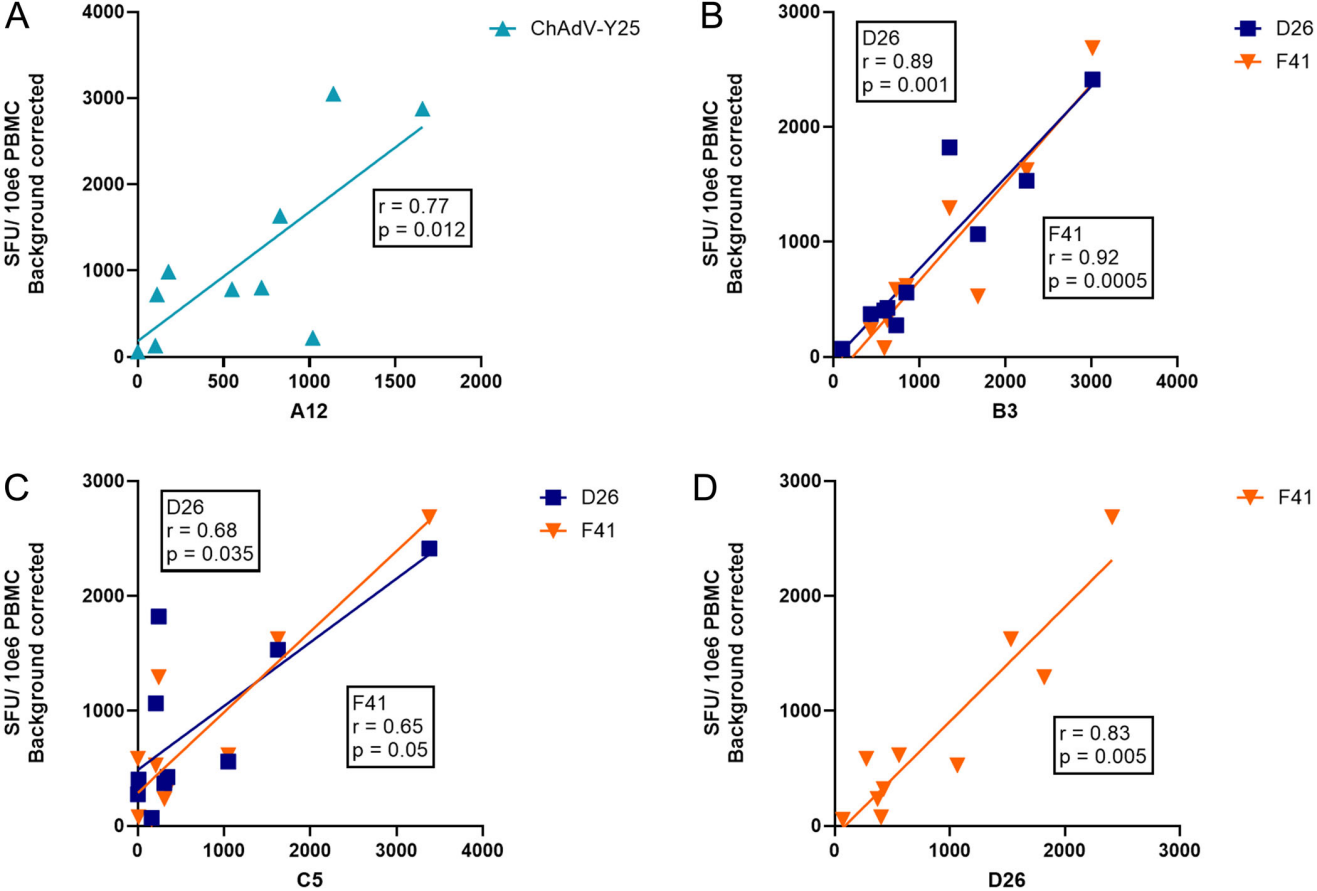
Plots showing the correlation between the frequency of IFNγ responses for pairs of hexons for ten healthy donors. Each axis shows the number of SFUs per 10^6^ PBMC produced by each donor in response to hexon peptide pool stimulation. Spearman's r correlation coefficients and two‐tailed p values are shown for statistically significant correlations.

There was a statistically significant correlation in the frequency of both IFNγ T cell responses (*r*2 = 0.24, *p* = 0.005) and IL2 responses to Y25 and C5 (*r*2 = 0.17, *p* = 0.018) (Supporting Information S1: Figure 5).

### 3.6 Relationship Between the Frequency of Cytokine Responses Between Species

Adenovirus‐specific T cell responses have previously been reported to be cross‐reactive within and between adenovirus species [9, 31]. Therefore, we hypothesised that cross‐reactive T cells from the same donor should recognise peptides from multiple different adenovirus species and that the magnitude of the response should be positively correlated: i.e., the IFNγ and IL2 response should be produced by a mixture of type or species‐specific T cells, and also a population of cross‐reactive T cells. Previous research suggests that the proportion of cross‐reactive T cells should be higher than the proportion of type/species‐specific T cells.

**Figure 6:**
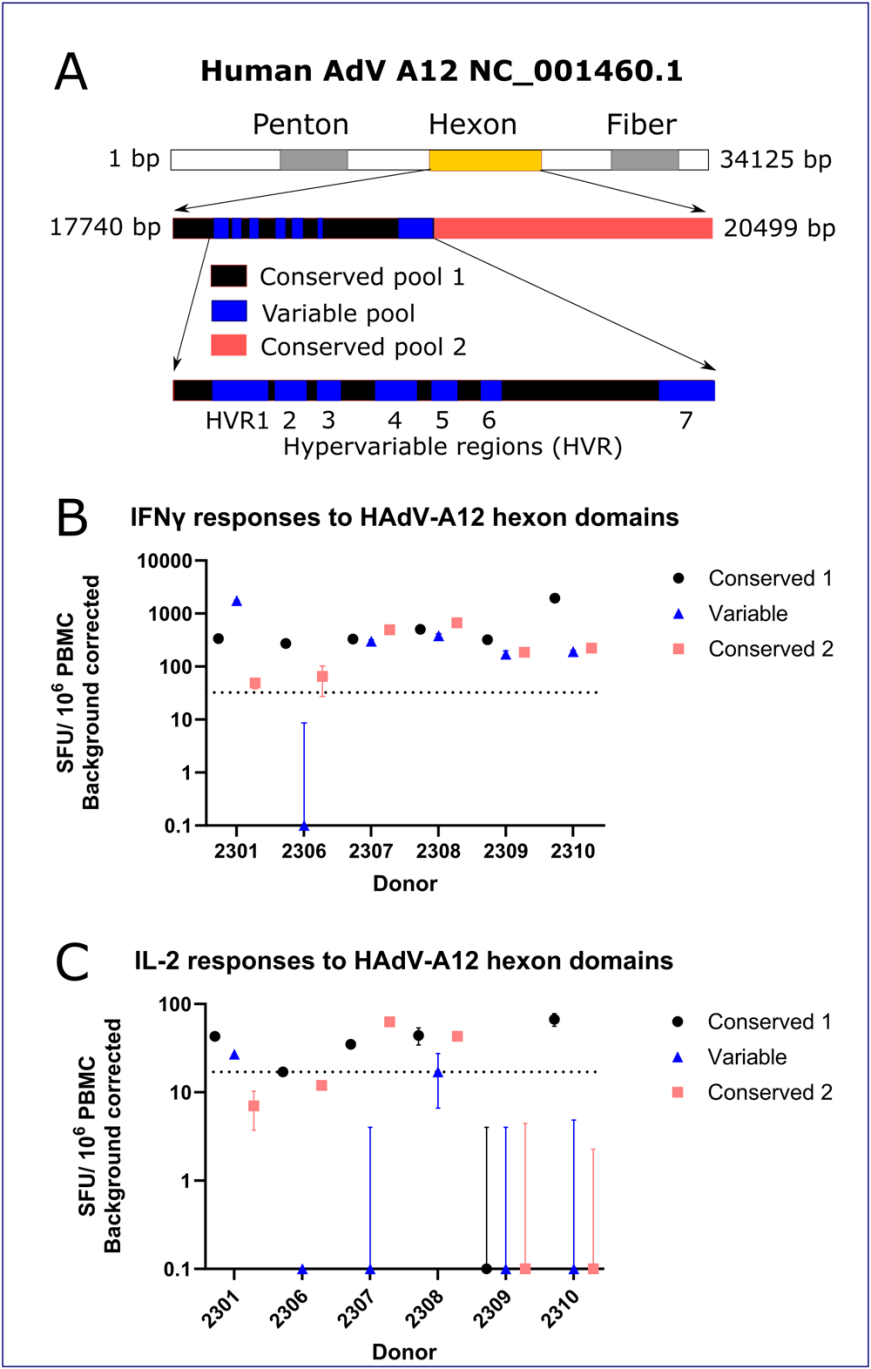
(A) Diagram showing the location of the adenovirus A12 hexon in genomic context, and the composition of the conserved and variable peptide pools derived from this protein. (B) Frequency of PBMC IFNγ responses to the HAdV‐A12 hexon, divided into two conserved and one variable epitope pool. Points show the mean (*n* = 3) and SEM for each donor and pool. The dotted line indicates a positive IFNγ response was defined as greater than 32.5 SFU/10^6^ PBMC (background corrected). (C) Frequency of PBMC IL2 responses to the conserved and variable epitope pools. The dotted line indicates a positive IL2 response was defined as greater than 17 SFU/10^6^ PBMC (background corrected).

We investigated this by calculating the correlations in the magnitude of SFU responses between each pair of hexon sequences. A number of pairs of hexon sequences had statistically significant Spearman's r coefficients for the frequency of IFNγ responses (Figure 5). There was a statistically significant relationship between the IFNγ and IL2 responses for genotypes A12 and Y25, B3 and D26, B3 and F41, and D26 and F41. IL2 response correlations are shown in the Supporting Information S1: Figure 6. Correlations between C5 and D26 and C5 and F41 were only significant for IFNγ responses.

Not all pairs of hexons had significantly correlated IFNγ and/or IL2 response frequencies. This suggests that the cross‐reactivity of adenovirus‐specific T cell responses may not apply equally across adenovirus species. Furthermore, a Mantel test of the correlation between genetic distances of paired hexon amino acid sequences, and the correlations in IFNγ responses between pairs of hexons, was not statistically significant. This suggests that shared infection history may explain the correlations between hexon pairs, rather than the degree of amino acid conservation.

### 3.7 Many Donors Make IFNγ T Cell Responses to Variable Regions of the Hexon

In adenovirus C, previous work had identified conserved regions of the hexon protein to be important for the T cell response, and in particular the CD4^+^ T cell response (e.g. [32]). To understand the relative contribution of variable and conserved regions of the hexon to the T cell response to adenovirus, the adenovirus A12 hexon was divided into three pools: one containing variable epitopes which are not highly conserved between adenovirus species, and two conserved pools, a 5' conserved pool 1 and a 3' conserved pool 2 (Supporting Information S1: Table 1) (Figure 6A). The variable pool included peptides which showed variation within species A genotypes. In a post‐SARS‐CoV‐2 emergence sample of donor serum from Germany, binding antibody responses to A12 were higher on average than responses to other species A genotypes, suggesting A12 was a suitable and seroprevalent representative of adaptive immune responses to species A AdVs in a post‐pandemic population [26].

We found the cellular immune response to A12 to include detectable IFNγ responses to all regions of the protein (Figure 6B), while the IL2 responses were more variable from donor to donor (Figure 6C). 5/6 healthy blood donors made an IFNγ response to the variable portion of the hexon, while all donors made an IFNγ response to the 5' and 3' conserved regions. The IL2 response to the variable domain was only present in 2/6 donors, in contrast to the IFNγ response. 5/6 donors made an IL2 response to 5' conserved pool 1, and 2/6 made responses to 3' conserved pool 2. This may reflect a bias of CD4^+^ T cell responses towards the more conserved regions of the hexon protein, which is less marked for CD8^+^ T cells.

## 4 Discussion

The de novo T cell response to adenovirus infection is widely recognised as ameliorating the severity of disease [33], while also playing a role in the success of adenovirus‐vectored vaccines and gene therapy products [34]. In this study, we investigated the frequency and function of adenovirus‐specific T cell responses in healthy donors using highly sensitive FluoroSpot assays, comparing responses to adenovirus proteins derived from five human and one chimpanzee adenovirus species. We also compared the effect of an adenovirus‐vectored vaccine (ChAdOx1) on T cell responses to the vector (ChAdV‐Y25) and a commonly used human adenovirus vector (HAdV‐C5).

We note a significant dichotomy between the high frequency of T cell responses to different adenovirus species (Figure 2) and the relatively small frequency of donors with detectable binding antibody levels to HAdV‐C5 (Figure 1). Recent data from healthy donors in Germany [26, 27] suggests that this may be because the majority of donors have low‐level circulating binding antibody responses to C5, represented by low OD values, while Schulze Lammers and colleagues used ELISpot to find that 138/151 donors have T cells which respond to either HAdV‐C5 hexon or penton peptide pools [35], which is similar to our findings. We speculate that ELISA‐based serological analysis of adenovirus binding antibody responses may not be sufficiently sensitive to establish past adenovirus infection history and immunity on a per‐genotype level. As with SARS‐CoV‐2, seroreversion may be a feature of infrequent adenovirus re‐exposure and/or reinfection in the adult population [23]. Additionally, the ‘hit and run’ strategy seen in these respiratory viruses may lead to low levels of serum antibodies in favour of mucosal responses dominated by IgA.

The commercial Peptivator AdV Select pool of peptides (Miltenyi) has been used for the generation of therapeutic anti‐HAdV T cell products by a number of groups e.g. [36, 37], and consists of a defined, experimentally validated set of HLA class I and II epitopes from HAdVs C2 and C5. We note that among healthy blood donors, more individuals made a detectable IL2 response to the HAdV C5 hexon pool than the AdV Select pool. The HAdV5 hexon peptide pool tiles the entire protein, and thus is agnostic to the HLA type of the donor. Indeed, both IFNγ and IL2 responses were statistically significantly more frequent to a peptide pool derived from the hexon of common respiratory HAdV B3 than to the AdV Select pool (Figure 2). This suggests that in donors of unknown HLA type, using a HAdV hexon pool which tiles the entire protein leads to more donors having a detectable IFNγ and IL2 T cell response than the AdV Select pool alone when generating therapeutic T cells for AdV cellular therapy.

Twenty‐five million first doses of the ChAdOx1 adenovirus‐vectored SARS‐CoV‐2 vaccine were administered to the UK's adult population received between 2021 and 2023 [19]. Y25 is the vector backbone from which ChAdOx1 was derived. In 2012, nAb responses to the vector were reported to be low in the UK population, with no donors having nAb titres where ND50 is at serum dilutions over 200 [21]; after immunisation with one or more doses of ChAdOx1, anti‐vector IgG responses became detectable [38]. Other studies have also identified that the use of a HAdV‐C5 vectored COVID vaccine boosts nAb responses in seronegative recipients [39]. Gardner and colleagues used T cell proliferation, cytokine secretion and intracellular flow cyotmetry assays to look at cross‐reactive T cell responses to the viral particles generated by the ChAdOx1 vector [40]. They found cross‐reactive T cell responses in vaccine naive and pre‐pandemic PBMC samples, which could be either to the vector backbone or the spike protein carried by the ChAdOx1 vector. Indeed, cross‐reactivity to spike has been described by many prior publications [23, 41, 42, 43]. In contrast, we focus on anti‐vector T cell responses to the structural proteins of backbone Y25 without additional SARS‐CoV‐2 antigens in the context of the UK's ChAdOx1 vaccination campaign. Among platelet donors at NHSBT Cambridge, from whom leucocyte reduction cones are derived, T cell responses to Y25 viral surface proteins are now ubiquitous and of comparable magnitude to common genotypes such as C5. In samples collected in 2021 from healthy donors, ChAdOx1 vaccine recipients had statistically significantly higher IFNγ responses to both Y25 and C5 hexons than controls, suggesting both a type‐specific and cross‐species boosting of cellular immunity to AdVs (Figure 4). This may have important consequences for future vaccination campaigns or gene therapy products wishing to use adenovirus vectors in individuals who have also received ChAdOx1.

Previous studies have shown that the hexon T cell response is equally distributed against both the variable and conserved regions of the protein [9] while others have suggested it is focused on conserved epitopes [10, 32]. Our data suggest that IFNγ responses, a proxy for CD8^+^ T cell responses [28], may be more skewed towards variable regions than previously thought (Figure 6B). The weak or absent correlation in the frequency of T cell responses to some pairs of hexons of different adenovirus species, and the common recognition of variable epitopes within the A12 hexon peptide pool by healthy blood donor PBMC, suggests an important species‐ or genotype‐specific component of the IFNγ response to adenovirus, which is therefore unlikely to be to conserved epitopes. There was no statistically significant correlation between amino acid distance between hexon pairs and the correlation in response frequency within donors, which suggests that shared infection history may explain the correlations in IFNγ responses to the genotypes of some hexon pairs. In contrast, IL2 responses (a proxy for the CD4^+^ T cell response [44]) to the variable domain of the A12 hexon peptide pool were relatively unusual (2/6 donors), which supports previous studies on the CD4^+^ T cell response being focused towards conserved epitopes (Figure 6C). The relative contribution of different classes of T cell response to adenovirus infection in adults merits further research to refine future vaccination and cellular therapy efforts.

## 5 Conclusions

We find that adenovirus‐specific cellular immune responses to five HAdV species are widespread in UK blood donors, and include inflammatory cytokine responses to a widely deployed SARS‐CoV‐2 vaccine vector backbone (ChAd‐Y25, used in the development of the ChAdOx1 vaccine). Responses to the penton protein are less commonly detected and at a lower frequency. We find that IFNγ responses to variable regions of the hexon protein may be more common than previously thought, particularly for genotype A12, while IL2 responses are often focused on conserved domains. We also present evidence that cross‐type and type‐specific IFNγ, but not IL2, responses have been boosted in ChAdOx1 recipients, with unknown consequences for population‐level immunity or future adenovirus evolution.

## Author Contributions

Charlotte J. Houldcroft conceived and designed the study. Charlotte J. Houldcroft, Benjamin J. Ravenhill and Charles N. Agoti supervised the study. Rookmini Mukhopadhyay, Arnold W. Lambisia, Jennifer P. Hoang, Benjamin A. Krishna and Charlotte J. Houldcroft performed the experiments and analysed the data. Jennifer P. Hoang, Charles N. Agoti, Benjamin A. Krishna, and Charlotte J. Houldcroft wrote the manuscript. All authors commented on the manuscript and approved submission.

## Conflicts of Interest

The authors declare no relevant conflicts of interest.

## Supporting information

Supporting information.

## Data Availability

The data that support the findings of this study are available on request from the corresponding author. The data are not publicly available due to privacy or ethical restrictions. The data are not publicly available due to privacy or ethical restrictions. Where not subject to privacy or ethical restrictions, the data that supports the findings of this study are available in the supplementary material of this article or are available on request from the corresponding author.
