# Supplementary material for: Adenovirus‐Specific T Cells in Adults Are Frequent, Cross‐Reactive to Common Childhood Adenovirus Infections and Boosted by Adenovirus‐Vectored Vaccines
Source: J Med Virol. 2025 Feb 8;97(2):e70222. doi: 10.1002/jmv.70222 (PMC11806872; doi:10.1002/jmv.70222)
Supplement: Supplementary file 1 — Supporting information. [file JMV-97-e70222-s001.docx]

**Title:** Adenovirus-specific T cells in adults are frequent, cross-reactive to common childhood adenovirus infections and boosted by adenovirus-vectored vaccines

Short title: AdV T cell cross-reactivity

**Authors**

Rookmini Mukhopadhyay1, Arnold W. Lambisia2, Jennifer P. Hoang1, Benjamin J. Ravenhill3, Charles N. Agoti2, Benjamin A. Krishna4, Charlotte J. Houldcroft1*

*Corresponding author: CJH [ch504@cam.ac.uk](mailto:ch504@cam.ac.uk)

**Supplementary methods**

PBMC and serum isolation

PBMC and serum were collected from leukocyte reduction system cones, a by-product of the platelet donation process. PBMC were separated from cones following a previously published protocol 20 using pluriSelect PBMC 24+ Spin Medium (Cambridge Bioscience, Cambridge, UK).

For both cohorts, PBMC were separated, frozen and thawed as previously described 21. Cell viability was determined using trypan blue exclusion staining and counting of live cells using a haemocytometer.

Phenotyping of PBMC

10^5 total PBMC were stained with the following phenotyping cocktail, containing 2 μl of each antibody: anti-CD3—fluorescein isothiocyanate (FITC), clone UCHT1; anti-CD4—phycoerythrin (PE), clone RPA-T4; anti-CD8a-peridinin-chlorophyll protein—cyanine 5.5 (PerCP Cy5.5), clone RPA-8a (all BioLegend, London, UK), and LIVE/DEAD Fixable Far Red Dead Cell Stain Kit (Thermo Fisher Scientific). Phenotyping and analysis were performed on the BD Accuri C6 flow cytometer.

Detection of Cytokine Production in PBMC by FluoroSpot

PBMC were incubated in pre-coated human IFNγ and IL2 FluoroSpot plates (Mabtech AB, Nacka Strand, Sweden) in duplicate (ChAdOx1 recipients and paired controls) or triplicate (healthy platelet donors) with ORF peptide pools (final peptide concentration shown in TABLE 1 following dilution with TexMacs) and an unstimulated and positive control mix [containing anti-CD3 and anti-CD28 (ImmunoCult Human CD3/CD28 T Cell Activator, StemCell)], for 48h at 37°C. Cells and media were decanted from the plate and developed following the manufacturer's protocol. After development and drying overnight, plates were read using an AID iSpot reader (Oxford Biosystems, Oxford, UK) and spot-forming units were counted using AID EliSpot v7 software (Autoimmun Diagnostika GmbH, Strasberg, Germany).

The mean spot forming units (SFU) were converted to SFU per 10^6 PBMC, the mean background response (SFU/10^6 cells) was deducted from the mean of the corresponding wells. The cutoff for a positive response was determined by comparing responses in known recipients of at least one dose of ChAdOx1 (5 × 10^10 viral particles per dose, equivalent to not less than 2.5 × 10^8 infectious units) versus donors who did not receive any doses of ChAdOx1. ROC analysis was conducted and determined to be 32.5 SFU/10^6 cells for IFNγ responses and 17 SFU/10^6 for IL2 responses, following the method in (Krishna et al., 2022). No clinical T cell correlate of protection is currently defined for human adenoviruses (Barnes et al., 2012; Koukoulias et al., 2023). Donors were excluded from further analysis if they failed to produce above-background IFNγ responses to positive control stimulation, compared to the negative control. Values at or below zero were plotted as 0.1 to allow their visualisation on logarithmic axes.

Power calculations

Sample size was computed using a two-sample t-test power calculation based on preliminary data of 8 donors, with a minimum difference of means of 7.78 and standard deviation of 5.85 for the unvaccinated group. This suggested that based on the difference between the means of two independent groups (unvaccinated vs vaccinated), that to achieve a type 1 error rate of 0.05 and power 90%, we would require a maximum of 12 members in each group; which was surpassed.

Multiple sequence alignment and amino acid distance calculations

Hexon amino acid sequences for HAdVs A12 (NP_040924.1), C5 (AP_000211.1), B3 (YP_002213779.1), D26 (ABO61316.1), F41 (ACH90432.1) and ChAd Y25 (YP_006272963.1) were retrieved from GenBank. Sequences were aligned in MEGA11 using MUSCLE and manually inspected. Estimated evolutionary distances between amino acid sequences were calculated in MEGA11 as the number of amino acid substitutions per site between sequences, with a Poisson correction model. Ambiguous positions were removed for each sequence pair using the pairwise deletion option. There were 976 positions in the final dataset.

**Supplementary figure 1**


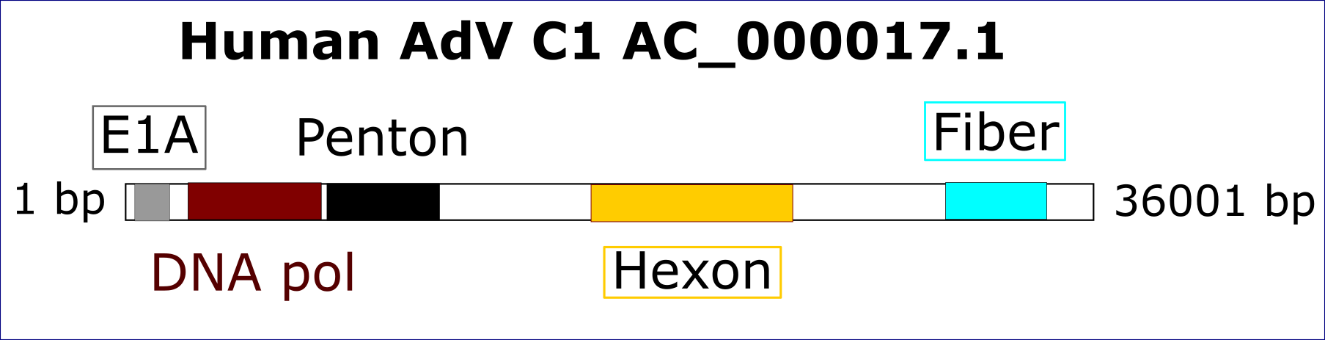


**Supplementary figure 1 legend**

Graphical representation of the human adenovirus C1 genome, highlighting selected adenovirus proteins which have evidence for human T cell responses. E1A (Keib et al., 2019), DNA polymerase (Joshi et al., 2009), and hexon (Leen et al., 2008; Olive et al., 2004) are all known to contain CD4+ and/or CD8+ T cell epitopes, while there is some evidence for penton and fiber T cell epitope recognition in a proportion of healthy blood donors (Tang et al., 2006). The neutralising antibody response is primarily targeted towards the hypervariable regions of the hexon protein (Toogood et al., 1992).

**Supplementary figure 2**


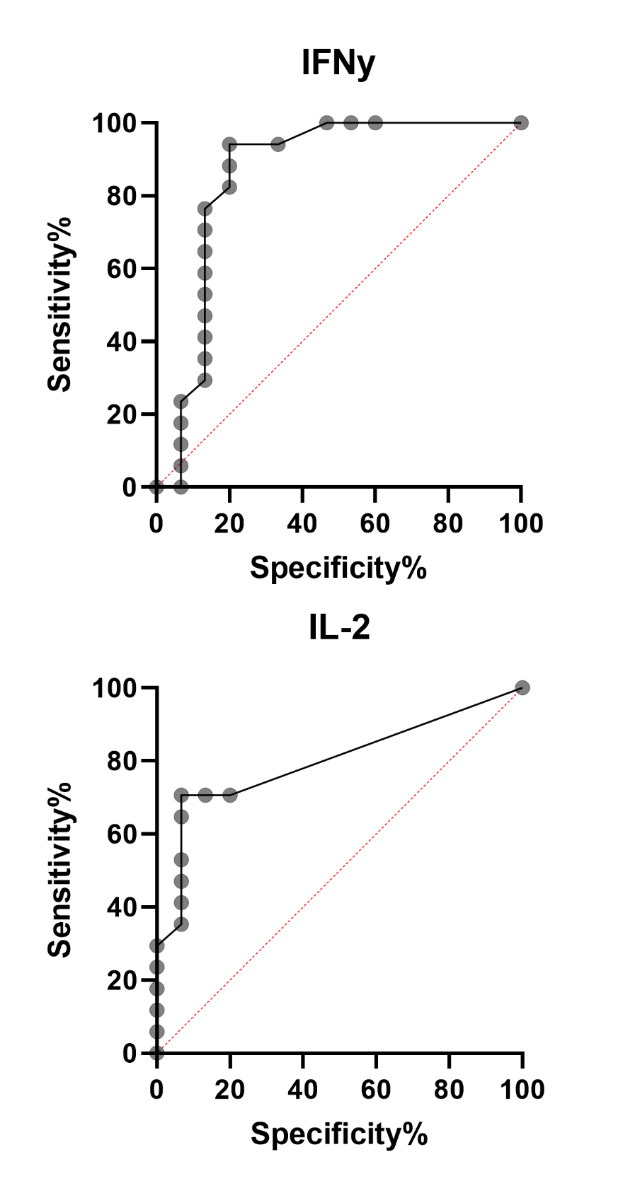


**Supplementary figure 2 legend**

Receiver operator curve analysis to determine optime positive/negative cutoff values for T cell Fluorospot responses to human adenovirus peptide pool stimulation. IFNγ and IL-2 responses to a Y25 peptide pool in known recipients and non-recipients (controls) ChAdOx1 were used.

**Supplementary figure 3**

**
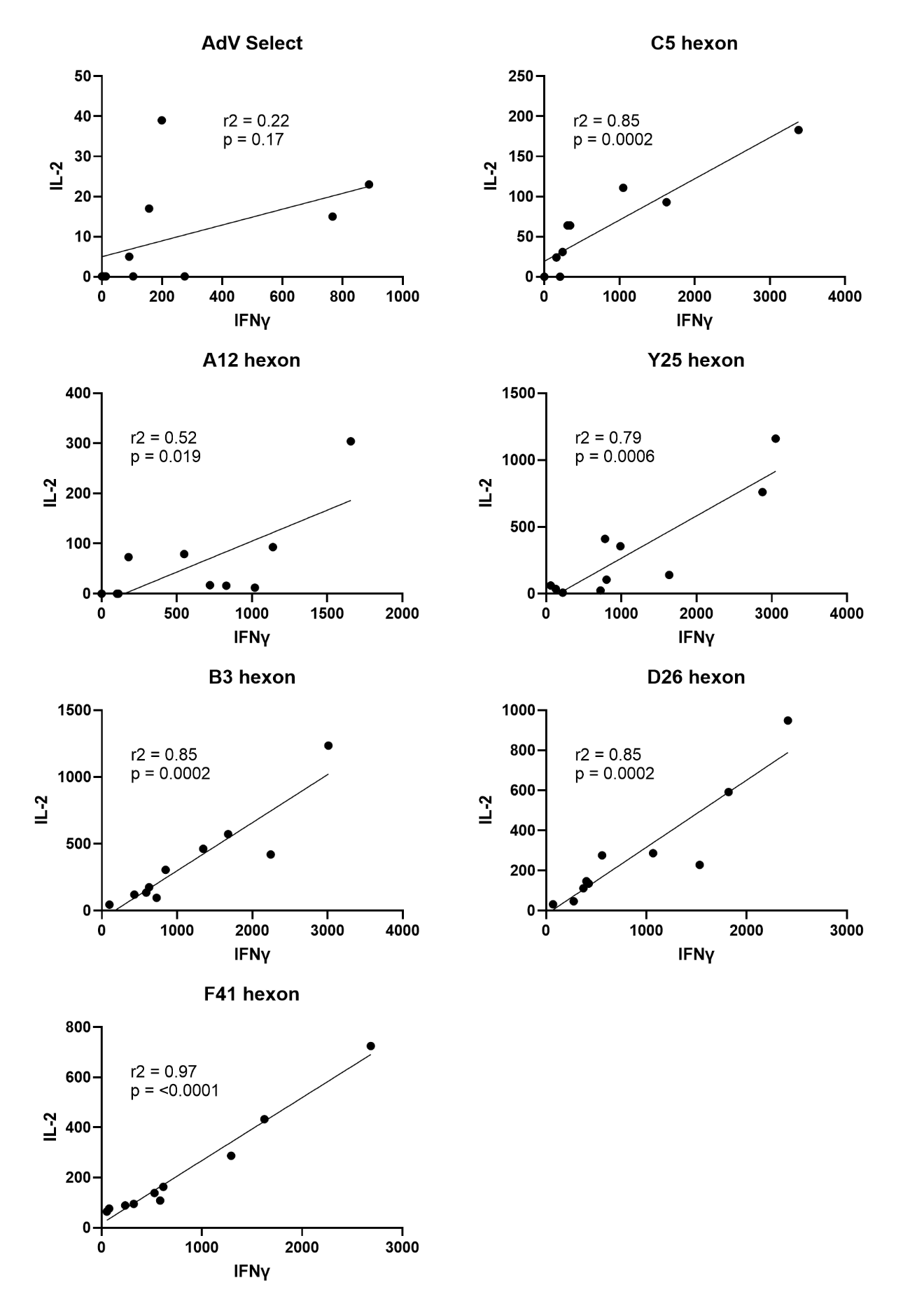
**

**Supplementary Figure 3 legend**

Plots showing within-donor correlations in background-corrected IFNγ and IL-2 responses, expressed as SFU per 10^6 PBMC. Spearman’s r correlation coefficients and two-tailed p values were calculated for each peptide pool.

**Supplementary figure 4**


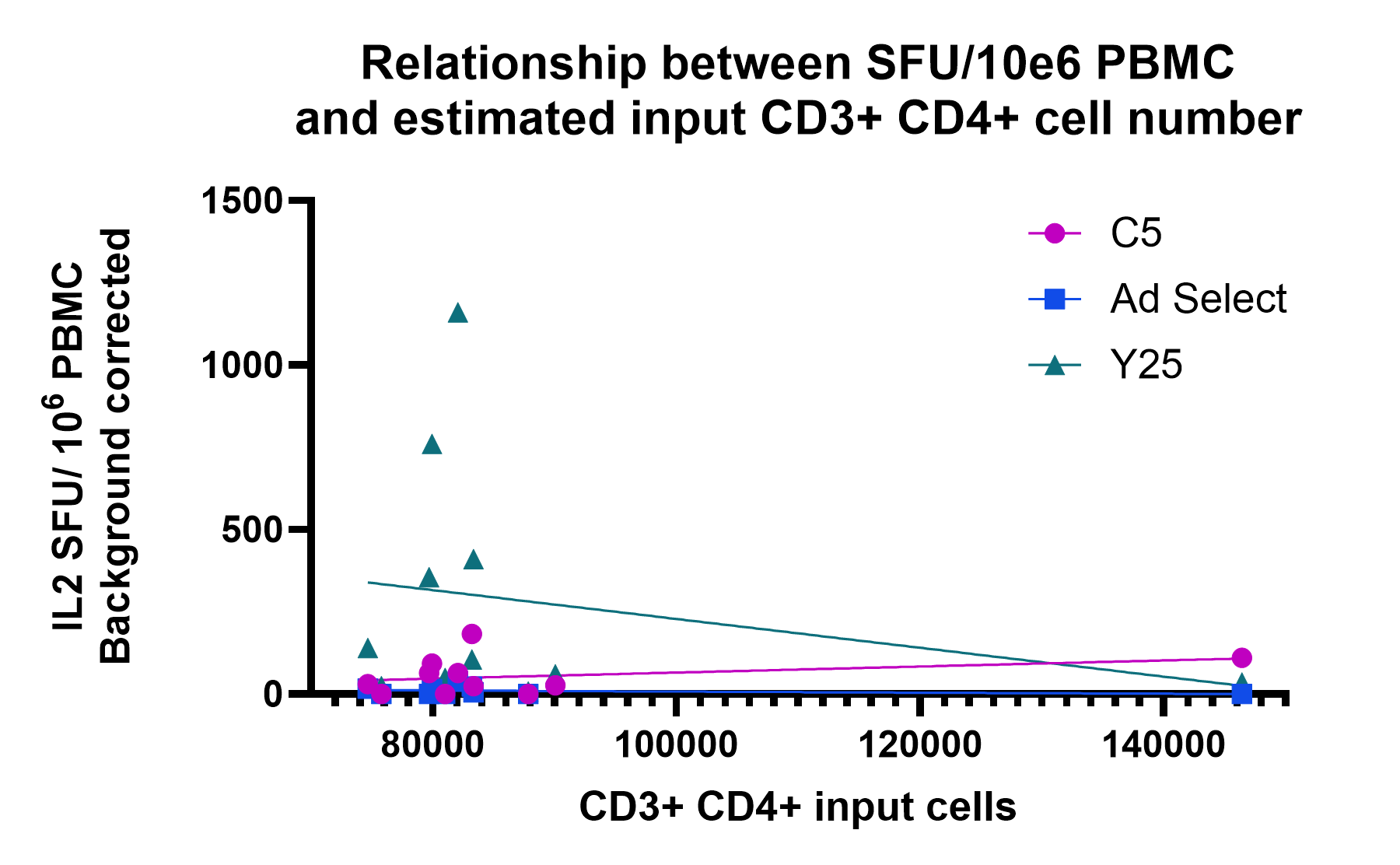
 **Supplementary Figure 4 legend**

Plot showing a simple linear regression of estimated input CD3+ CD4+ lymphocytes per donor, and the IL2 SFU per 10^6^ PBMC (corrected for background). A simple linear regression was performed in Graphpad Prism 10.3 for peptide pools C5, Y25 and Peptivator Ad Select. None of the slopes were significantly non-zero.

**Supplementary figure 5**

**
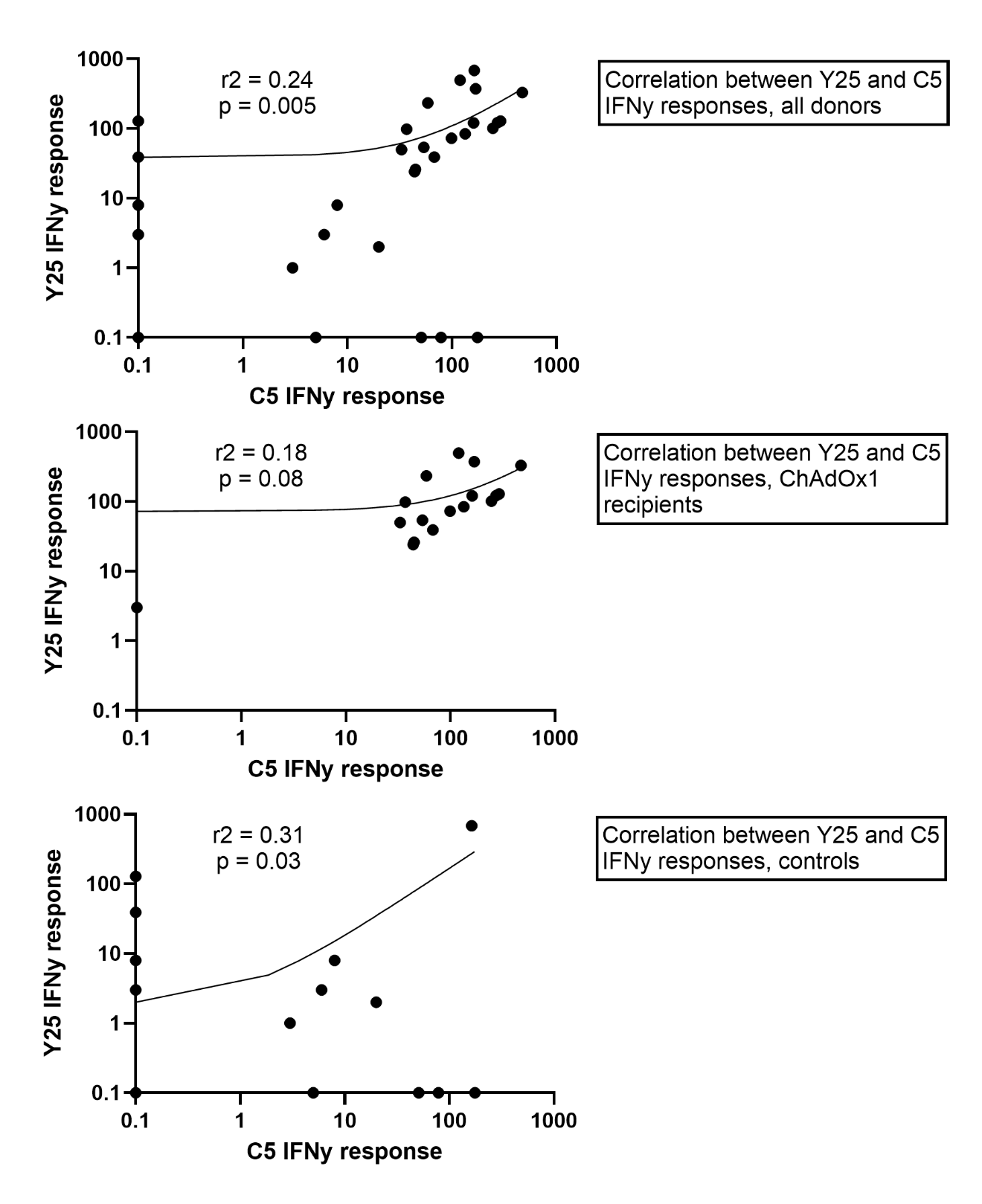
**

**A**


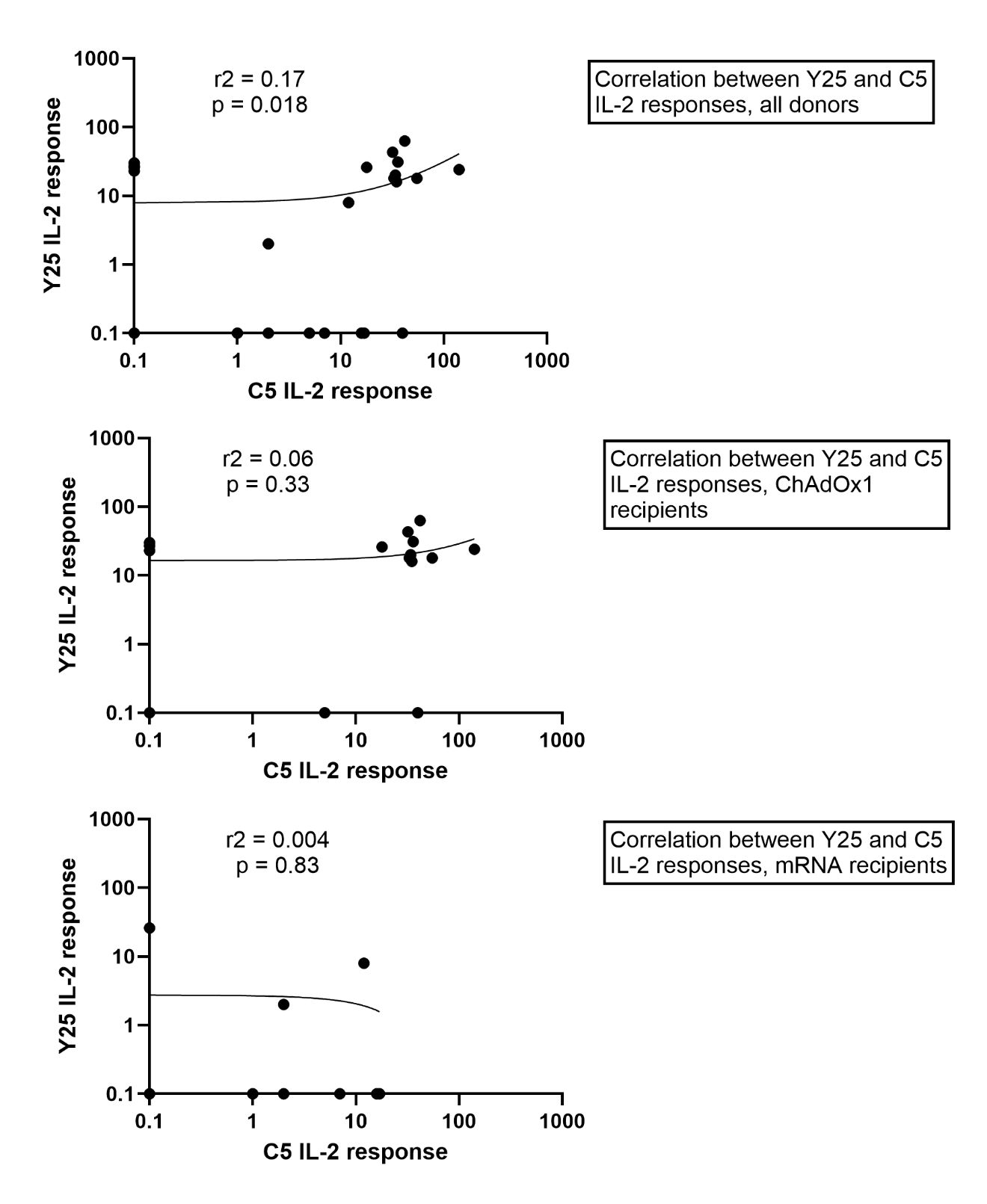


**B**

**Supplementary Figure 5 legend**

Simple linear regression of frequency of (A) IFNγ and (B) IL-2 T cell responses to hexon peptide pools derived from AdV Y25 and C5. Regressions were performed on all donors, on ChAdOx1 recipients only and on controls only. There was a statistically significant positive correlation in the all donors group for both IFNγ and IL-2.

**Supplementary figure 6**


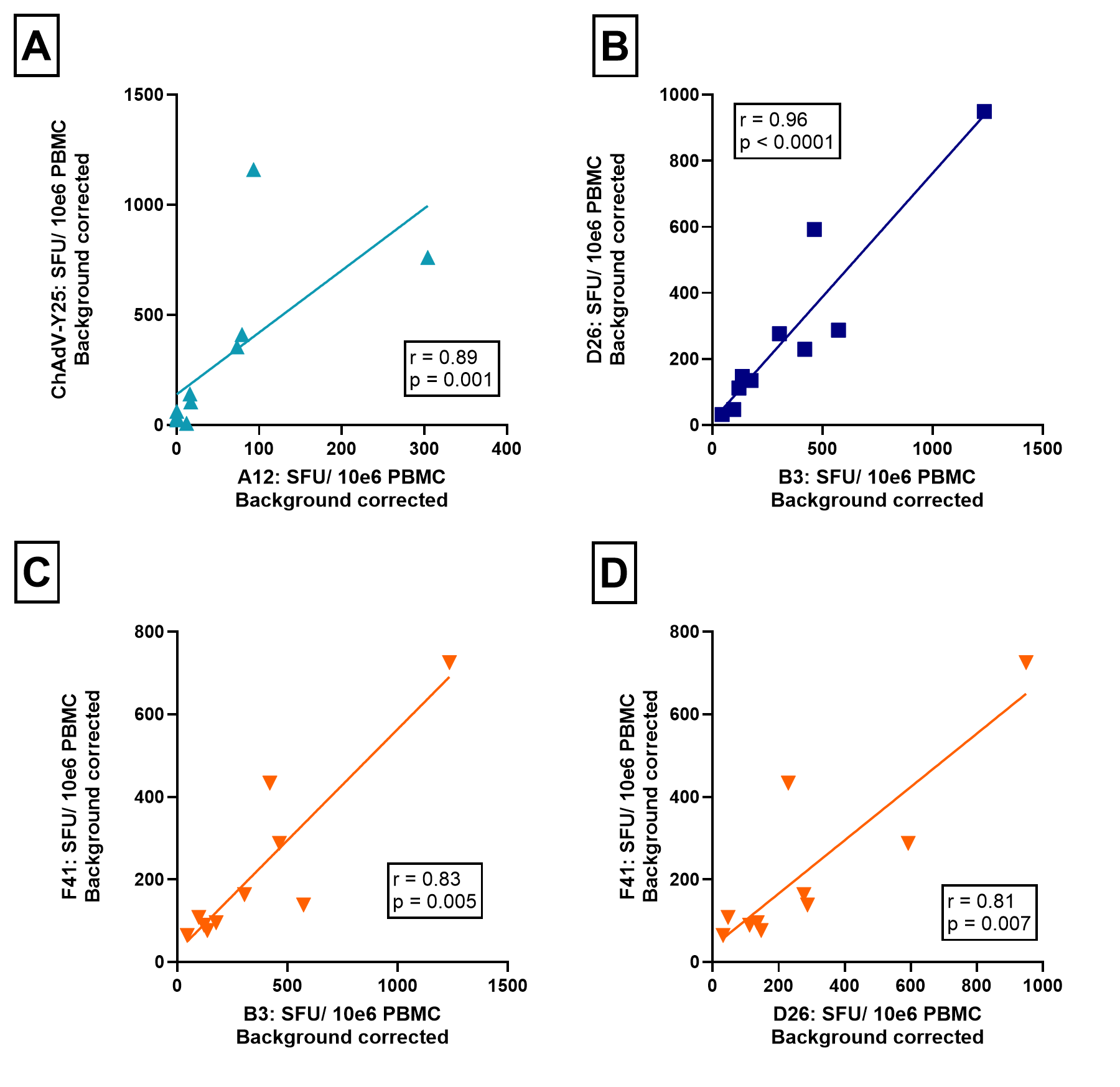


**Supplementary figure 6 legend**

Plots showing the correlation between the frequency of IL2 responses, expressed as spot-forming units per 10^6^ PBMC (background corrected), for pairs of hexons. A shows correlations between HAdV-A12 and ChAd-Y25; B shows the correlation between HAdV B3 and D26; C shows the correlation between HAdVs D26 and F41; D shows ; B shows the correlation between HAdV B3 and F41. Spearman’s r correlation coefficients and two-tailed p values shown for statistically significant correlations.

**Supplementary table 1**

The epitopes constituting the A12 conserved 1, conserved 2 and variable pools.

| Number | Sequence | Pool |
| --- | --- | --- |
| 1 | MATPSMMPQWSYMHI | Conserved 1 |
| 2 | SYMHIAGQDASEYLS | Conserved 1 |
| 3 | SEYLSPGLVQFARAT | Conserved 1 |
| 4 | FARATDTYFTLGNKF | Conserved 1 |
| 5 | LGNKFRNPTVAPTHD | Conserved 1 |
| 6 | APTHDVTTDRSQRLT | Conserved 1 |
| 7 | SQRLTLRFVPVDRED | Conserved 1 |
| 8 | VDREDTTYSYKARFT | Conserved 1 |
| 9 | KARFTLAVGDNRVLD | Conserved 1 |
| 10 | NRVLDMASSYFDIRG | Conserved 1 |
| 11 | FDIRGVLDRGPSFKP | Conserved 1 |
| 12 | PSFKPYSGTAYNSLA | Conserved 1 |
| 13 | YNSLAPKGAPNASQW | Conserved 1 |
| 14 | NASQWSDNAKLNTFA | Variable |
| 15 | LNTFAQAPYLSDTIT | Variable |
| 16 | SDTITAADGIKVGTD | Variable |
| 17 | KVGTDTAQAGAAVYA | Variable |
| 18 | AAVYANKTYQPEPQV | Variable |
| 19 | PEPQVGPSEWNTSIE | Variable |
| 20 | NTSIENVKAGGRALK | Variable |
| 21 | GRALKQTTAMQPCYG | Variable |
| 22 | QPCYGSYARPTNEHG | Variable |
| 23 | TNEHGGQSKDDNIEL | Variable |
| 24 | DNIELKFFDSANNAA | Variable |
| 25 | ANNAANTAQVVFYTE | Variable |
| 26 | VFYTEDVNLEMPDTH | Variable |
| 27 | MPDTHLVFKPTVTNG | Variable |
| 28 | TVTNGTIASESLLGQ | Variable |
| 29 | SLLGQQAAPNRANYI | Variable |
| 30 | RANYIAFRDNFIGLM | Conserved 1 |
| 31 | FIGLMYYNSTGNMGV | Conserved 1 |
| 32 | GNMGVLAGQASQLNA | Conserved 1 |
| 33 | SQLNAVVDLQDRNTE | Conserved 1 |
| 34 | DRNTELSYQLMLDAL | Conserved 1 |
| 35 | MLDALGDRTRYFSLW | Conserved 1 |
| 36 | YFSLWNSAVDSYDPD | Conserved 1 |
| 37 | SYDPDVRVIENHGVE | Conserved 1 |
| 38 | NHGVEDELPNYCFPL | Conserved 1 |
| 39 | YCFPLSAVGEIKNYK | Variable |
| 40 | IKNYKGIKPDNGGGG | Variable |
| 41 | NGGGGGWTADNTVSE | Variable |
| 42 | NTVSEANHIGIGNIA | Variable |
| 43 | IGNIAAMEINLQANL | Variable |
| 44 | LQANLWRSFLYSNVG | Variable |
| 45 | YSNVGLYLPDDLKYT | Variable |
| 46 | DLKYTPGNIKLPDNK | Variable |
| 47 | LPDNKNTYEYMNGRV | Variable |
| 48 | MNGRVTAPGLVDTYV | Variable |
| 49 | VDTYVNIGARWSPDV | Variable |
| 50 | WSPDVMDNVNPFNHH | Conserved 1 |
| 51 | PFNHHRNAGLRYRSM | Conserved 1 |
| 52 | RYRSMLLGNGRFVPF | Conserved 1 |
| 53 | RFVPFHIQVPQKFFA | Conserved 1 |
| 54 | QKFFAIRNLLLLPGS | Conserved 1 |
| 55 | LLPGSYTYEWNFRKD | Conserved 1 |
| 56 | NFRKDVNMILQSTLG | Conserved 1 |
| 57 | QSTLGNDLRVDGASV | Conserved 1 |
| 58 | DGASVRFDNIALYAN | Conserved 1 |
| 59 | ALYANFFPMAHNTAS | Conserved 1 |
| 60 | HNTASTLEAMLRNDT | Conserved 2 |
| 61 | LRNDTNDQSFNDYLC | Conserved 2 |
| 62 | NDYLCAANMLYPIPA | Conserved 2 |
| 63 | YPIPANATSVPISIP | Conserved 2 |
| 64 | PISIPSRNWAAFRGW | Conserved 2 |
| 65 | AFRGWSFTRLKTKET | Conserved 2 |
| 66 | KTKETPSLGSGFDPY | Conserved 2 |
| 67 | GFDPYFVYSGTIPYL | Conserved 2 |
| 68 | TIPYLDGTFYLNHTF | Conserved 2 |
| 69 | LNHTFKKVSIMFDSS | Conserved 2 |
| 70 | MFDSSVSWPGNDRLL | Conserved 2 |
| 71 | NDRLLTPNEFEIKRS | Conserved 2 |
| 72 | EIKRSVDGEGYNVAQ | Conserved 2 |
| 73 | YNVAQCNMTKDWFLI | Conserved 2 |
| 74 | DWFLIQMLSHYNIGY | Conserved 2 |
| 75 | YNIGYQGFYIPESYK | Conserved 2 |
| 76 | PESYKDRMYSFFRNF | Conserved 2 |
| 77 | FFRNFQPMSRQVVDT | Conserved 2 |
| 78 | QVVDTTEYKNYKKVT | Variable |
| 79 | YKKVTVEFQHNNSGF | Conserved 2 |
| 80 | NNSGFVGYLGPTMRE | Conserved 2 |
| 81 | PTMREGQAYPANYPY | Conserved 2 |
| 82 | ANYPYPLIGQTAVES | Conserved 2 |
| 83 | TAVESITQKKFLCDR | Conserved 2 |
| 84 | FLCDRVMWRIPFSSN | Conserved 2 |
| 85 | PFSSNFMSMGALTDL | Conserved 2 |
| 86 | ALTDLGQNMLYANSA | Conserved 2 |
| 87 | YANSAHALDMTFEVD | Conserved 2 |
| 88 | TFEVDPMDEPTLLYV | Conserved 2 |
| 89 | TLLYVLFEVFDVVRI | Conserved 2 |
| 90 | DVVRIHQPHRGVIEA | Conserved 2 |
| 91 | GVIEAVYLRTPFSAG | Conserved 2 |

**References**

Barnes, E., Folgori, A., Capone, S., Swadling, L., Aston, S., Kurioka, A., Meyer, J., Huddart, R., Smith, K., Townsend, R., Brown, A., Antrobus, R., Ammendola, V., Naddeo, M., O’Hara, G., Willberg, C., Harrison, A., Grazioli, F., Esposito, M. L., … Klenerman, P. (2012). Novel adenovirus-based vaccines induce broad and sustained T cell responses to HCV in man. *Science Translational Medicine*, *4*(115). https://doi.org/10.1126/SCITRANSLMED.3003155/SUPPL_FILE/4-115RA1_SM.PDF

Joshi, A., Tang, J., Kuzma, M., Wagner, J., Mookerjee, B., Filicko, J., Carabasi, M., Flomenberg, N., & Flomenberg, P. (2009). Adenovirus DNA polymerase is recognized by human CD8+ T cells. *Journal of General Virology*, *90*(1), 84–94. https://doi.org/10.1099/VIR.0.002493-0/CITE/REFWORKS

Keib, A., Mei, Y.-F., Cicin-Sain, L., Busch, D. H., & Dennehy, K. M. (2019). Measuring Antiviral Capacity of T Cell Responses to Adenovirus. *The Journal of Immunology*, *202*(2), 618–624. https://doi.org/10.4049/JIMMUNOL.1801003

Koukoulias, K., Papayanni, P. G., Jones, J., Kuvalekar, M., Watanabe, A., Velazquez, Y., Gilmore, S., Papadopoulou, A., Leen, A. M., & Vasileiou, S. (2023). Assessment of the cytolytic potential of a multivirus-targeted T cell therapy using a vital dye-based, flow cytometric assay. *Frontiers in Immunology*, *14*, 1299512. https://doi.org/10.3389/FIMMU.2023.1299512/BIBTEX

Krishna, B. A., Lim, E. Y., Mactavous, L., Lyons, P. A., Doffinger, R., Bradley, J. R., Smith, K. G. C., Sinclair, J., Matheson, N. J., Lehner, P. J., Wills, M. R., & Sithole, N. (2022). Evidence of previous SARS-CoV-2 infection in seronegative patients with long COVID. *EBioMedicine*, *81*. https://doi.org/10.1016/J.EBIOM.2022.104129

Leen, A. M., Christin, A., Khalil, M., Weiss, H., Gee, A. P., Brenner, M. K., Heslop, H. E., Rooney, C. M., & Bollard, C. M. (2008). Identification of Hexon-Specific CD4 and CD8 T-Cell Epitopes for Vaccine and Immunotherapy. *Journal of Virology*, *82*(1), 546–554. https://doi.org/10.1128/JVI.01689-07/ASSET/6C1DD73F-4834-4980-BF2F-6006F0541F11/ASSETS/GRAPHIC/ZJV0010800830005.JPEG

Olive, M., Eisenlohr, L., Flomenberg, N., Hsu, S., & Flomenberg, P. (2004). The Adenovirus Capsid Protein Hexon Contains a Highly Conserved Human CD4+ T-Cell Epitope. *Https://Home.Liebertpub.Com/Hum*, *13*(10), 1167–1178. https://doi.org/10.1089/104303402320138952

Tang, J., Olive, M., Pulmanausahakul, R., Schnell, M., Flomenberg, N., Eisenlohr, L., & Flomenberg, P. (2006). Human CD8+ cytotoxic T cell responses to adenovirus capsid proteins. *Virology*, *350*(2), 312–322. https://doi.org/10.1016/J.VIROL.2006.01.024

Toogood, C. I. A., Crompton, J., & Hay, R. T. (1992). Antipeptide antisera define neutralizing epitopes on the adenovirus hexon. *Journal of General Virology*, *73*(6), 1429–1435. https://doi.org/10.1099/0022-1317-73-6-1429/CITE/REFWORKS
